# Branched-chain amino acid and branched-chain ketoacid ingestion increases muscle protein synthesis rates in vivo in older adults: a double-blind, randomized trial

**DOI:** 10.1093/ajcn/nqz120

**Published:** 2019-06-28

**Authors:** Cas J Fuchs, Wesley J H Hermans, Andrew M Holwerda, Joey S J Smeets, Joan M Senden, Janneau van Kranenburg, Annemie P Gijsen, Will K H W Wodzig, Henk Schierbeek, Lex B Verdijk, Luc J C van Loon

**Affiliations:** 1 Department of Human Biology, NUTRIM School of Nutrition and Translational Research in Metabolism, Maastricht University Medical Centre+, Maastricht, Netherlands; 2 Central Diagnostic Laboratory, Maastricht University Medical Centre+, Maastricht, Netherlands; 3 Department of Pediatrics, Emma Children's Hospital, Academic Medical Center, Amsterdam, Netherlands

**Keywords:** aging, sarcopenia, chronic kidney disease, anabolism, milk, dietary protein, leucine, α-ketoisocaproic acid

## Abstract

**Background:**

Protein ingestion increases muscle protein synthesis rates. However, limited data are currently available on the effects of branched-chain amino acid (BCAA) and branched-chain ketoacid (BCKA) ingestion on postprandial muscle protein synthesis rates.

**Objective:**

The aim of this study was to compare the impact of ingesting 6 g BCAA, 6 g BCKA, and 30 g milk protein (MILK) on the postprandial rise in circulating amino acid concentrations and subsequent myofibrillar protein synthesis rates in older males.

**Methods:**

In a parallel design, 45 older males (age: 71 ± 1 y; BMI: 25.4 ± 0.8 kg/m^2^) were randomly assigned to ingest a drink containing 6 g BCAA, 6 g BCKA, or 30 g MILK. Basal and postprandial myofibrillar protein synthesis rates were assessed by primed continuous l-[ring-^13^C_6_]phenylalanine infusions with the collection of blood samples and muscle biopsies.

**Results:**

Plasma BCAA concentrations increased following test drink ingestion in all groups, with greater increases in the BCAA and MILK groups compared with the BCKA group (*P* < 0.05). Plasma BCKA concentrations increased following test drink ingestion in all groups, with greater increases in the BCKA group compared with the BCAA and MILK groups (*P* < 0.05). Ingestion of MILK, BCAA, and BCKA significantly increased early myofibrillar protein synthesis rates (0–2 h) above basal rates (from 0.020 ± 0.002%/h to 0.042 ± 0.004%/h, 0.022 ± 0.002%/h to 0.044 ± 0.004%/h, and 0.023 ± 0.003%/h to 0.044 ± 0.004%/h, respectively; *P <* 0.001), with no differences between groups (*P* > 0.05). Myofibrillar protein synthesis rates during the late postprandial phase (2–5 h) remained elevated in the MILK group (0.039 ± 0.004%/h; *P* < 0.001), but returned to baseline values following BCAA and BCKA ingestion (0.024 ± 0.005%/h and 0.024 ± 0.005%/h, respectively; *P >* 0.05).

**Conclusions:**

Ingestion of 6 g BCAA, 6 g BCKA, and 30 g MILK increases myofibrillar protein synthesis rates during the early postprandial phase (0–2 h) in vivo in healthy older males. The postprandial increase following the ingestion of 6 g BCAA and BCKA is short-lived, with higher myofibrillar protein synthesis rates only being maintained following the ingestion of an equivalent amount of intact milk protein. This trial was registered at Nederlands Trial Register (www.trialregister.nl) as NTR6047.

## Introduction

Protein ingestion strongly increases muscle protein synthesis rates (1, 2). The postprandial increase in muscle protein synthesis rate has been attributed to the rise in circulating amino acids (3, 4). Amino acids serve as precursors for de novo muscle protein synthesis and can act as strong signaling molecules activating translation initiation via the mechanistic/mammalian target of rapamycin complex-1 (mTORC1) pathway (5, 6). Several studies indicate that senescent muscle is less sensitive to these anabolic properties of amino acids (7, 8). Anabolic resistance to feeding has been reported in the elderly and in several patient populations suffering from chronic disease (9). As a consequence, older and/or more clinically compromised patient populations require greater amounts of protein to be consumed (7, 10–14) or may benefit from food fortification with branched-chain amino acids (BCAAs) (15–19) to augment postprandial muscle protein synthesis rates. Limited data are available regarding the muscle protein synthetic response to ingesting BCAAs (leucine, isoleucine, and valine) only in humans. Recently, it was shown that BCAA ingestion increases myofibrillar protein synthesis rates during recovery from exercise in young males (20). However, whether BCAA ingestion can increase myofibrillar protein synthesis rates to a similar extent when compared with the ingestion of intact protein in vivo in older males remains to be assessed.

In many clinically compromised populations, simply increasing protein intake is not realistic and has been suggested to be unfavorable in patients with chronic kidney disease (CKD) because of potential renal injury (21). Hence (very) low protein diets are often prescribed in certain disease stages of patients with CKD, further compromising their capacity to preserve muscle mass (22–24). Supplementation with branched-chain ketoacids (BCKAs) has been applied in these conditions as these keto-analogs do not provide nitrogen (N) and may help to lower nitrogen intake as BCKAs can be transaminated into BCAAs (25, 26). BCKAs are readily available, safe for human consumption (27), and efficiently absorbed in the small intestine (28). However, intestinal absorption rates of BCKAs appear to be moderately lower when compared with BCAAs (29). In addition, oral administration of BCKAs, but not BCAAs, appears to induce substantial first-pass oxidation in splanchnic organs (30). Therefore, the nutritional efficiency, and thus bioavailability of ingested BCKAs, may be considerably lower when compared with BCAAs (26, 31). A lower bioavailability may suggest that BCKAs do not stimulate muscle protein synthesis to a similar extent when compared with BCAAs or intact protein. Furthermore, it has been observed that intravenous infusion of BCKAs does not stimulate whole-body protein synthesis (32). However, it is important to note that whole-body protein synthesis is not necessarily reflective of muscle protein synthesis and evidence from animal work in fact supports a role for BCKAs as a nutrient regulator of muscle protein synthesis (33, 34). To date, no studies have investigated the effects of ingesting BCKAs on muscle protein synthesis in humans. We hypothesize that ingestion of intact protein, BCAAs, as well as BCKAs stimulates myofibrillar protein synthesis in vivo in older males.

## Methods

### Subjects

Forty-five healthy (tracer naïve) older men (age: 71 ± 1 y; BMI: 25.4 ± 0.8 kg/m^2^) participated in this double-blind, parallel-group, randomized trial. The trial was conducted between January 2017 and May 2017 at Maastricht University Medical Centre+, in Maastricht, The Netherlands (for the consort flow chart, please see **Supplemental Figure 1**). The characteristics of the subjects are detailed in **Table 1**. All subjects were informed of the purpose of the study, experimental procedures, and possible risks before providing written consent to participate. The procedures followed were in accordance with the ethical standards of the Medical Ethics Committee of the Maastricht University Medical Centre+ on human experimentation and in accordance with the Helsinki Declaration of 1975 as revised in October 2013. This trial was registered at www.trialregister.nl as NTR6047.

**TABLE 1:** Subjects’ characteristics^1^

	BCAA	BCKA	MILK	*P*
Age, y	70 ± 1	71 ± 1	72 ± 1	0.201
Body mass, kg	78.5 ± 2.2	79.0 ± 3.2	80.7 ± 2.2	0.779
BMI, kg/m^2^	25.9 ± 0.9	25.4 ± 0.6	24.9 ± 0.8	0.568
Systolic BP, mmHg	138 ± 3	134 ± 4	131 ± 3	0.345
Diastolic BP, mmHg	72 ± 1	68 ± 3	66 ± 2	0.101
Fat, %	24.4 ± 1.6	24.1 ± 0.9	21.9 ± 1.6	0.297
Appendicular lean mass, kg	25.3 ± 0.8	26.1 ± 0.9	26.5 ± 0.9	0.558
Lean body mass, kg	57.2 ± 1.5	57.6 ± 2.2	60.7 ± 1.5	0.246
Fasting glucose (OGTT), mmol/L	5.2 ± 0.2	4.9 ± 0.2	5.0 ± 0.2	0.346
2-h glucose (OGTT), mmol/L	7.2 ± 0.9	5.6 ± 0.4	5.8 ± 0.6	0.103
Fasting insulin (OGTT), mU/L	10.8 ± 1.8	10.1 ± 1.7	10.0 ± 1.1	0.915
2-h insulin (OGTT), mU/L	74.0 ± 20.9	59.1 ± 19.1	39.9 ± 10.0	0.307
HbA1c, %	5.5 ± 0.2	5.5 ± 0.1	5.5 ± 0.1	0.986
HOMA2-IR	1.4 ± 0.2	1.3 ± 0.2	1.3 ± 0.2	0.913
OGIS, mL/min/m^2^	367 ± 21	385 ± 19	409 ± 19	0.252

^1^Values represent means ± SEM, *n* = 15 per group. Data were analyzed using a one-factor ANOVA. No differences were detected between groups. BCAA, 6 g branched-chain amino acids; BCKA, 6 g branched-chain ketoacids; BP, blood pressure; HbA1c, glycated hemoglobin; HOMA2-IR, homeostatic model assessment of insulin resistance; MILK, 30 g milk protein; OGIS, oral glucose insulin sensitivity; OGTT, oral-glucose-tolerance test.

### Ethics approval and consent to participate

This study was approved by the Medical Ethical Committee of the Maastricht University Medical Centre+, The Netherlands (METC 16-3-035). All participants provided written informed consent before participation.

### Pretesting

Volunteers between the age of 65–80 y and with a BMI between 18.5 and 30.0 underwent medical screening to assess glycated hemoglobin (HbA1c), whole-body glucose tolerance [using a 2-h oral-glucose-tolerance test (35)], blood pressure, weight, height, and body composition (by DXA; Discovery A; Hologic). Subjects were deemed healthy based on their responses to a medical questionnaire and their screening results.

### Study design

Subjects were randomly assigned to consume a drink containing 30 g milk protein (MILK) (ReFit MPI 85; Friesland Campina; *n* = 15), 6 g BCAAs (Evonik Industries; *n* = 15), or 6 g BCKAs (Myolution; Evonik Industries; *n* = 15). The 30 g MILK provides ∼6 g BCAAs with a total of 2.64 g leucine, which should theoretically induce a measurable increase in postprandial muscle protein synthesis rates ( 36). The ratio of (keto-)leucine, (keto-)isoleucine, and (keto-)valine in both BCAA and BCKA drinks was 2:1:1. Hence, the BCAA and BCKA drinks provided 3 g (keto-)leucine, 1.5 g (keto-)isoleucine, and 1.5 g (keto-)valine (total 6 g). Randomization was performed by using a computerized random-number generator. An independent person was responsible for random assignment and drink preparation.

### Diet and physical activity

All subjects were instructed to refrain from any sort of strenuous physical activity 3 d prior to the infusion trial and to keep their diet as consistent as possible for 2 d prior to the experiment. On the evening before the experimental trial, all subjects consumed the same standardized meal (2061 kJ/487 kcal) providing 31.8 g protein, 58.7 g carbohydrate, and 11.3 g fat at ∼1800 h followed by an evening snack (985 kJ/234 kcal) composed of 34.7 g protein, 18.5 g carbohydrate, and 0.0 g fat at 2200 h.

### Infusion protocol

**FIGURE 1:**
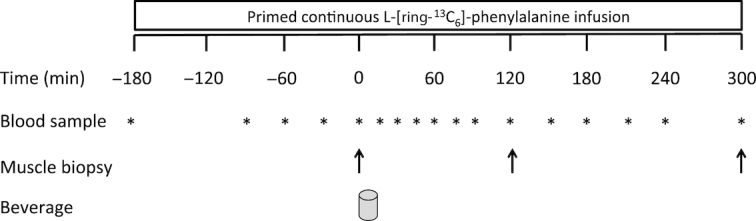
Schematic overview of the infusion protocol. Participants consumed either 30 g MILK, 6 g BCAAs, or 6 g BCKAs.

At 0800 h, after an overnight fast, subjects arrived at the laboratory by car or public transport. A catheter was inserted into an antecubital vein for stable isotope labeled amino acid infusion. A second catheter was inserted into a dorsal hand vein of the contralateral arm and placed in a hot box (60°C) for arterialized blood sampling (37). After obtaining a baseline blood sample, the plasma phenylalanine pools were primed with a single dose of l-[ring-^13^C_6_]-phenylalanine (2.25 μmol·kg^−1^), after which a continuous l-[ring-^13^C_6_]-phenylalanine (0.05 μmol·kg^−1^·min^−1^) intravenous infusion was initiated (*t* = −180 min). Subsequently, the subjects rested in a supine position for 180 min during which 4 additional arterialized blood samples were drawn (*t* = −90, −60, −30, and 0 min). A muscle biopsy sample was then collected from the m. vastus lateralis of a randomly chosen leg (*t* = 0 min). After collection of the first muscle biopsy sample, subjects consumed a drink containing 30 g MILK (*n* = 15), 6 g BCAAs (*n* = 15), or 6 g BCKAs (*n* = 15) at *t* = 0 min. A small amount of l-[ring-^13^C_6_]-phenylalanine (6%) was added to the MILK beverage to prevent precursor pool dilution. Additional arterialized blood samples were collected at *t* = 15, 30, 45, 60, 75, 90, 120, 150, 180, 210, 240, and 300 min. Second and third muscle biopsies were collected at *t* = 120 min and *t* = 300 min to determine postprandial myofibrillar protein synthesis rates. Blood samples were collected in EDTA-containing tubes and centrifuged at 1000*× g* for 10 min at 4°C. Aliquots of plasma were snap frozen in liquid nitrogen and stored at −80°C. Biopsy samples were collected from the middle region of the M. vastus lateralis, ∼15 cm above the patella and 3 cm below entry through the fascia, using the percutaneous needle biopsy technique (38). Muscle samples were dissected carefully, freed from any visible nonmuscle material, immediately frozen in liquid nitrogen, and stored at −80°C until further analysis. For a schematic representation of the infusion protocol, see **Figure 1**.

### Plasma and muscle tissue analysis

Details of analysis related to the determination of plasma (glucose, insulin, ammonia, ketoacids, amino acids, HbA_1c_, l-[ring-^13^C_6_]-phenylalanine, mixed plasma proteins) as well as muscle (myofibrillar protein l-[ring*-*^13^C_6_]-phenylalanine enrichments) data are presented in the **Supplemental Methods**.

### Calculations

Myofibrillar protein fractional synthetic rates (FSRs) were calculated using the standard precursor-product equation, as follows (39):

(1) $$\begin{equation*}
FSR = \frac{{\Delta {E_p}}}{{{E_{precursor}} \cdot t}} \cdot 100
\end{equation*}$$

Δ*E_p_* is the increment in myofibrillar protein-bound l-[ring-^13^C_6_]-phenylalanine enrichment after an incorporation period, *E*_precursor_ is the weighted mean plasma l-[ring-^13^C_6_]-phenylalanine enrichment during that incorporation period, and *t* is the incorporation period (h). Weighted mean plasma enrichments were calculated by taking the average enrichment between all consecutive time points and correcting for the time between these sampling time points. The weighted mean plasma precursor pool is preferred in this setting because the more frequent sampling time points allow a more accurate correction of the transient changes in precursor pool enrichments over time (40). For basal FSR, plasma protein samples at *t* = −180 min and muscle biopsy samples at *t* = 0 min were used; and for postprandial FSRs, muscle biopsy samples at *t* = 0, 120, and 300 min were used.

### Statistical analysis

All data are expressed as means ± SEM. Baseline characteristics, incremental AUC (iAUC; for postprandial plasma insulin and ammonia concentrations), and plasma amino acid enrichments were compared between treatment groups using one-factor ANOVA. For time-dependent variables, repeated-measures ANOVA with treatment as a between-subjects factor and time as a within-subjects factor was used (i.e., all time points for plasma concentrations and basal compared with postprandial for muscle data). In case of significant interactions, separate analyses were performed within each treatment group, as well as between treatment groups for every time period separately (e.g., for FSR values, separate one-factor ANOVA for basal, 0–2 h, 2–5 h, and 0–5 h). In the case of significant treatment effects, Bonferroni post hoc analyses were performed to locate the effects. Significance was set at *P* < 0.05. Calculations were performed using SPSS (version 21.0, IBM Corp.).

## Results

### Plasma glucose, insulin, and ammonia

**FIGURE 2:**
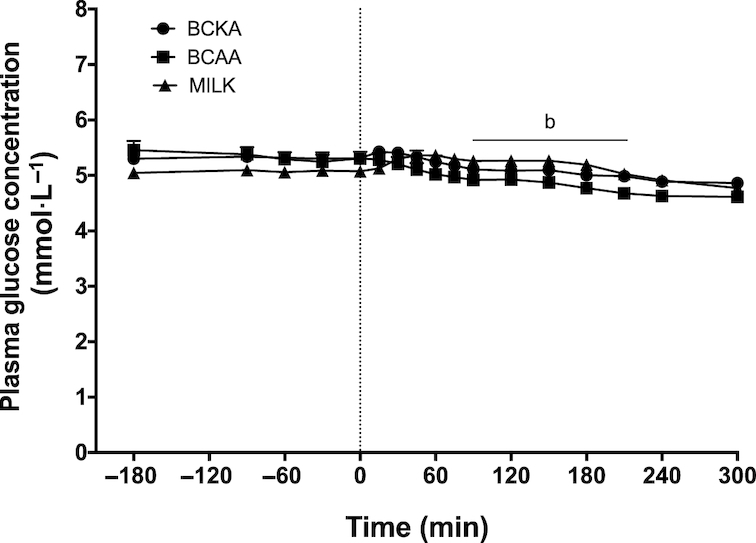
Plasma glucose concentrations over time after the ingestion of 30 g milk protein (MILK; *n* = 15), 6 g branched-chain amino acids (BCAA; *n* = 15), or 6 g branched-chain ketoacids (BCKA; *n* = 15) in healthy older males. The dotted line represents the ingestion of the drink. Values represent means + SEM. Data were analyzed with repeated measures (time × treatment group) ANOVA and separate analyses were performed when a significant interaction was detected. Bonferroni post hoc testing was used to detect differences between groups. Time × treatment interaction, *P* < 0.001. b, MILK significantly different (*P* < 0.05) from BCAA.

**FIGURE 3:**
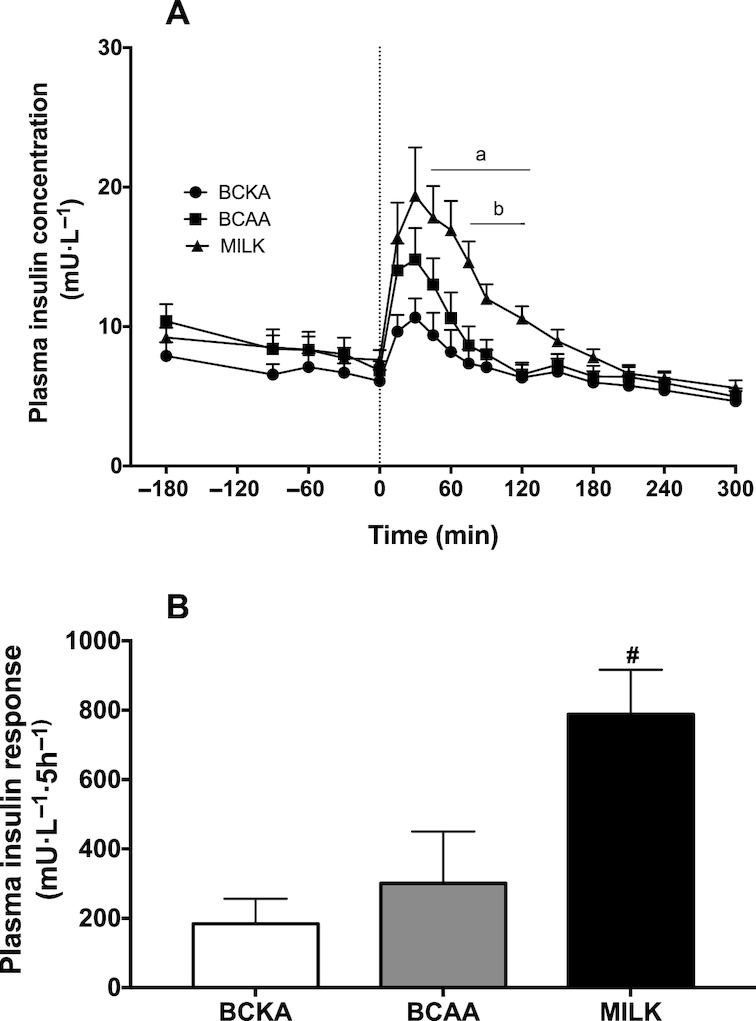
Plasma insulin concentrations over time (A) and total insulin responses, expressed as incremental AUC (B) after the ingestion of 30 g milk protein (MILK; *n* = 15), 6 g branched-chain amino acids (BCAA; *n* = 15), or 6 g branched-chain ketoacids (BCKA; *n* = 15) in healthy older males. The dotted line represents the ingestion of the drink. Values represent means + SEM. Data in panel A were analyzed using repeated measures (time × treatment group) ANOVA and separate analyses were performed when a significant interaction was detected. Bonferroni post hoc testing was used to detect differences between groups. Time × treatment interaction, *P* < 0.001. a, MILK significantly different (*P* < 0.05) from BCKA; b, MILK significantly different (*P* < 0.05) from BCAA. Data in panel B were analyzed using a one-factor ANOVA with Bonferroni correction. ^#^Significantly different (*P* < 0.05) from BCAA and BCKA.

**FIGURE 4:**
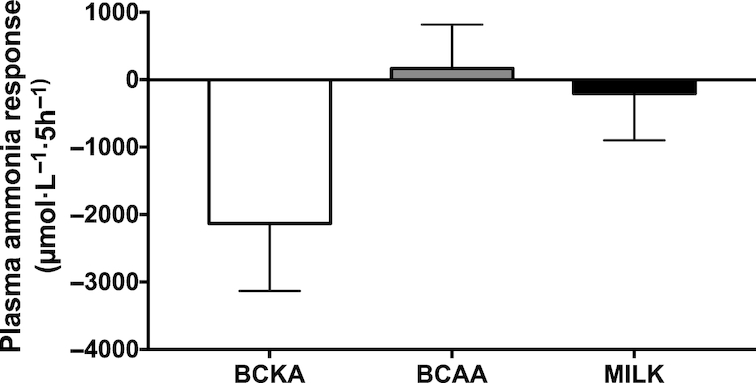
Plasma total ammonia concentrations, expressed as incremental AUC after the ingestion of 30 g milk protein (MILK; *n* = 15), 6 g branched-chain amino acids (BCAA; *n* = 15), or 6 g branched-chain ketoacids (BCKA; *n* = 15) in healthy older males. Values represent means ± SEM. Data were analyzed using a one-factor ANOVA. No significant differences between treatments (*P* > 0.05).

Plasma glucose concentrations (**Figure 2**) slightly declined over time in the BCKA and BCAA groups (*P* < 0.05), whereas they slightly increased over time in the MILK group (*P* < 0.05). Plasma glucose concentrations were significantly higher between *t* = 90 and 210 min in the MILK group compared with the BCAA group (*P* < 0.05). Plasma insulin concentrations (**Figure 3**A) showed a rapid and significant increase following beverage ingestion in the BCAA and MILK group (*P* < 0.05), but not in the BCKA group. A greater increase in plasma insulin concentrations was observed in the MILK group compared with the BCAA group between *t* = 75 and 120 min (*P* < 0.05). In agreement, the iAUC above fasting plasma insulin concentrations was significantly greater in the MILK group compared with the BCKA and BCAA groups (*P* < 0.05; Figure 3B). The iAUC of plasma ammonia (**Figure 4**) did not show significant differences between groups, despite the fact that plasma ammonia concentrations were substantially reduced during the postprandial period in the BCKA group.

### Plasma amino acids

**FIGURE 5:**
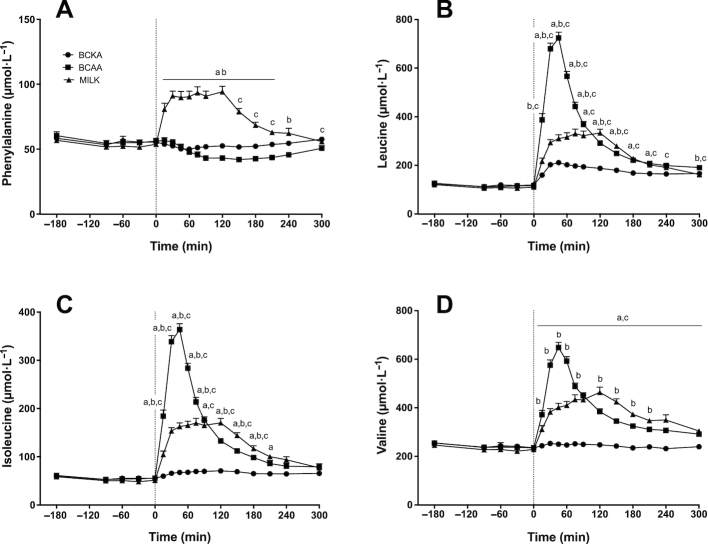
Plasma phenylalanine (A), leucine (B), isoleucine (C), and valine (D) concentrations over time after the ingestion of 30 g milk protein (MILK; *n* = 15), 6 g branched-chain amino acids (BCAA; *n* = 15), or 6 g branched-chain ketoacids (BCKA; *n* = 15) in healthy older males. The dotted line represents the ingestion of the drink. Values represent means + SEM. Data were analyzed with repeated measures (time × treatment group) ANOVA and separate analyses were performed when a significant interaction was detected. Bonferroni post hoc testing was used to detect differences between groups. Time × treatment interaction, *P* < 0.001. a, MILK significantly different (*P* < 0.05) from BCKA; b, MILK significantly different (*P* < 0.05) from BCAA; c, BCAA significantly different (*P* < 0.05) from BCKA.

Plasma phenylalanine (**Figure 5**A), leucine (Figure 5B), isoleucine (Figure 5C), and valine (Figure 5D) concentrations over time are depicted in Figure 5. Significant time × treatment interactions were observed for plasma phenylalanine, leucine, isoleucine, and valine concentrations (*P* < 0.001). Following drink ingestion, a rapid increase in plasma phenylalanine concentrations was found in the MILK group (*P* < 0.05), whereas plasma phenylalanine concentrations remained unchanged and decreased over time in the BCKA and BCAA groups, respectively (*P* < 0.05). Plasma leucine concentrations increased significantly after drink ingestion in all groups (*P* < 0.01), with the highest peak plasma leucine concentrations measured in the BCAA group (*P* < 0.05). Plasma isoleucine and valine concentrations increased significantly after drink ingestion and remained elevated for the entire postprandial period in the BCAA and MILK groups (*P* < 0.01), whereas no changes were observed in the BCKA group.

**FIGURE 6:**
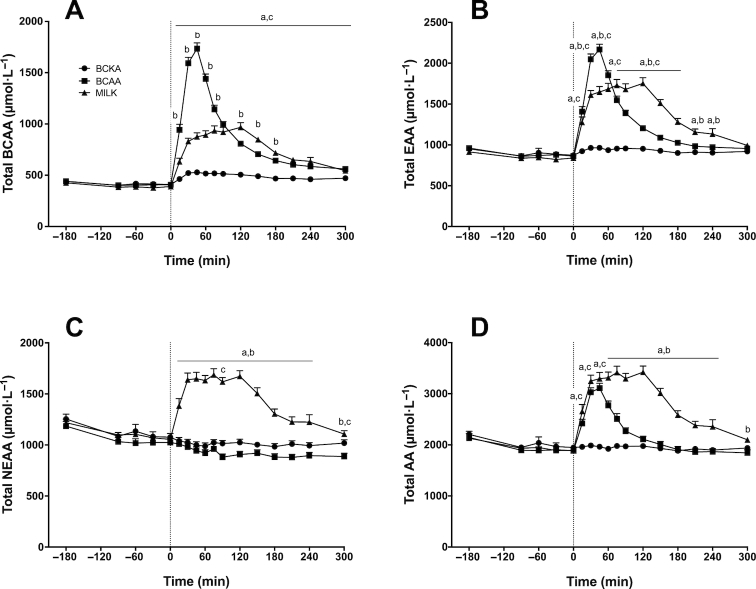
Plasma branched-chain amino acids (BCAA; A), essential amino acid (EAA; B), nonessential amino acid (NEAA-glutamine; C), and total amino acid (TAA-glutamine; D) concentrations over time after the ingestion of 30 g milk protein (MILK; *n* = 15), 6 g BCAA (*n* = 15), or 6 g branched-chain ketoacids (BCKA; *n* = 15) in healthy older males. The dotted line represents the ingestion of the drink. Values represent means + SEM. Data were analyzed with repeated measures (time × treatment group) ANOVA and separate analyses were performed when a significant interaction was detected. Bonferroni post hoc testing was used to detect differences between groups. Time × treatment interaction, *P* < 0.001. a, MILK significantly different (*P* < 0.05) from BCKA; b, MILK significantly different (*P* < 0.05) from BCAA; c, BCAA significantly different (*P* < 0.05) from BCKA.

Plasma BCAA (**Figure 6**A), essential amino acid (EAA; Figure 6B), nonessential amino acid (NEAA-glutamine; Figure 6C), and total amino acid (TAA; Figure 6D) concentrations over time are depicted in Figure 6. For BCAA, EAA, NEAA, and TAA concentrations, significant time × treatment interactions were observed (*P* < 0.001). Plasma total BCAA concentrations increased significantly after drink ingestion and remained elevated for the entire postprandial period in the BCAA and MILK groups (*P* < 0.001). For the BCKA group, plasma total BCAA concentrations were only significantly higher than fasting values at *t* = 150 and 300 min (*P* < 0.01). Plasma EAA concentrations increased significantly after drink ingestion and remained elevated for the entire postprandial period in the BCAA (apart from *t* = 240 min; *P* > 0.05) and MILK groups (*P* < 0.05), whereas no increase was found after BCKA ingestion. Plasma total NEAA concentrations following drink ingestion significantly increased and remained elevated until *t* = 240 min after MILK ingestion (*P* < 0.001), did not change after BCKA ingestion, and were significantly lower when compared to fasting values at *t* = 90, 180, 210, and 300 min in the BCAA group (*P* < 0.05). Plasma TAA concentrations immediately increased after BCAA (until *t* = 120 min; *P* < 0.001) and MILK ingestion (remained elevated for the entire postprandial period; *P* < 0.01). No significant changes were observed after BCKA ingestion.

### Plasma α-ketoacids

**FIGURE 7:**
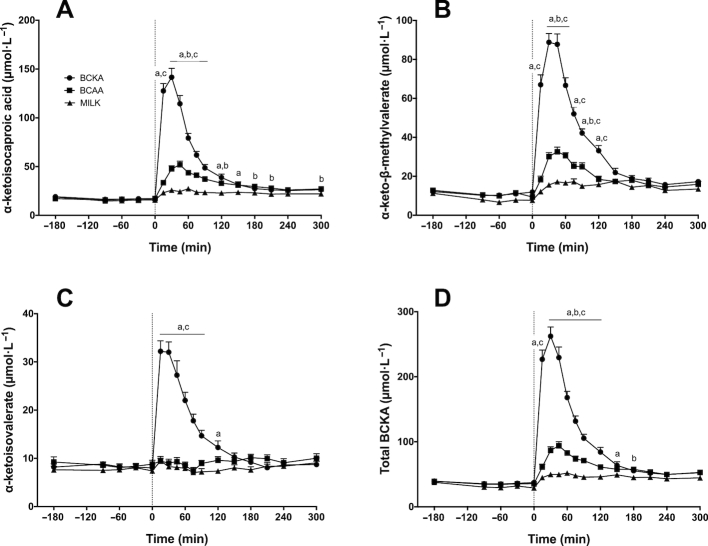
Plasma α-ketoisocaproic acid (KIC; the α-ketoacid of leucine; A), α-keto-β-methylvalerate (KMV; the α-ketoacid of isoleucine; B), α-ketoisovalerate (KIV; the α-ketoacid of valine; C), and branched-chain ketoacid (BCKA) (D) concentrations over time after the ingestion of 30 g milk protein (MILK; *n* = 15), 6 g branched-chain amino acids (BCAA; *n* = 15), or 6 g BCKA (*n* = 15) in healthy older males. The dotted line represents the ingestion of the drink. Values represent means + SEM. Data were analyzed with repeated measures (time × treatment group) ANOVA and separate analyses were performed when a significant interaction was detected. Bonferroni post hoc testing was used to detect differences between groups. Time × treatment interaction, *P* < 0.001. a, MILK significantly different (*P* < 0.05) from BCKA; b, MILK significantly different (*P* < 0.05) from BCAA; c, BCAA significantly different (*P* < 0.05) from BCKA.

Plasma α-ketoisocaproic acid (KIC; the α-ketoacid of leucine; **Figure 7**A), α-keto-β-methylvalerate (KMV; the α-ketoacid of isoleucine; Figure 7B), α-ketoisovalerate (KIV; the α-ketoacid of valine; Figure 7C), and total BCKA (Figure 7D) concentrations over time are depicted in Figure 7. Significant time × treatment interactions were observed for plasma KIC, KMV, KIV, and total BCKA concentrations (*P* < 0.001). Following drink ingestion, plasma KIC and KMV concentrations significantly increased and remained elevated for the entire postprandial period in the BCKA (*P* < 0.001, apart from *t* = 240 min for KMV) and BCAA groups (from *t* = 30 min; *P* < 0.05). For the MILK group, KIC concentrations were only significantly higher than fasting values between *t* = 150–300 min and KMV concentrations were only significantly higher than fasting values between *t* = 75–210 min and at *t* = 300 min (*P* < 0.05). Following drink ingestion, plasma KIV concentrations significantly increased in the BCKA group (from *t* = 15 until *t* = 150 min; *P* < 0.01), whereas no changes were observed in the BCAA and MILK groups. Plasma total BCKA concentrations increased significantly after drink ingestion for the entire postprandial period in the BCKA (*P* < 0.001) and BCAA groups (*P* < 0.001; apart from *t* = 15 min), whereas they were only significantly increased between *t* = 90–300 min in the MILK group (*P* < 0.05).

### Isotope tracer analysis

Prior to BCAA, BCKA, or MILK ingestion plasma l-[ring-^13^C_6_]-phenylalanine enrichments averaged 7.5 ± 0.3, 7.4 ± 0.1, and 7.4 ± 0.2 mole % excess (MPE), respectively, with no differences between treatments (data not shown). Plasma l-[ring-^13^C_6_]-phenylalanine enrichments during the postprandial period averaged 8.7 ± 0.3, 7.9 ± 0.2, and 7.3 ± 0.2 MPE for the BCAA, BCKA, and MILK groups respectively, with significant differences between the BCAA and BCKA groups (*P* < 0.01) as well as the BCAA and MILK groups (*P* < 0.001), but not between the BCKA and MILK groups (*P* = 0.082).

**FIGURE 8:**
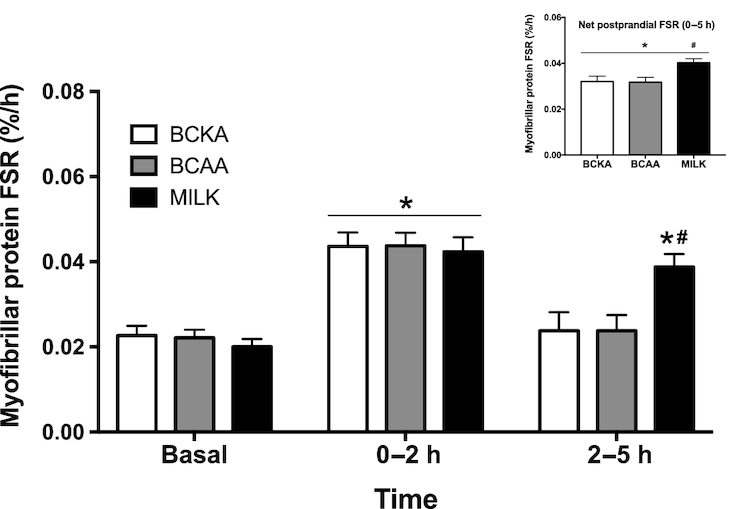
Myofibrillar protein fractional synthetic rates (FSR; in %/h) during the fasting state (basal) and over the early (0–2 h), late (2–5 h), and entire (0–5 h) postprandial period after the ingestion of 30 g milk protein (MILK; *n* = 15), 6 g branched-chain amino acids (BCAA; *n* = 15), or 6 g branched-chain ketoacids (BCKA; *n* = 15) in healthy older males. Values represent means + SEM. Data were analyzed with repeated measures (time × treatment group) ANOVA and separate analyses were performed when a significant interaction was detected. Bonferroni post hoc testing was used to detect differences between groups. *Significantly different (*P* < 0.01) from basal; ^#^significantly different (*P* < 0.05) from BCAA and BCKA.

Myofibrillar protein synthesis rates calculated based on the plasma precursor pool are depicted in **Figure 8**. No differences were observed in basal muscle protein synthesis rates between groups (*P =* 0.624). Myofibrillar protein synthesis rates increased from basal to the early (0–2 h) postprandial period in all groups (from 0.020 ± 0.002%/h to 0.042 ± 0.004%/h in the MILK group, 0.022 ± 0.002%/h to 0.044 ± 0.004%/h in the BCAA group, and 0.023 ± 0.003%/h to 0.044 ± 0.004%/h in the BCKA group; *P <* 0.001), with no differences between treatment groups (time × treatment interaction: *P* = 0.969, main treatment effect: *P* = 0.732). A significant time × treatment interaction (*P =* 0.002) showed that after ingestion of MILK, myofibrillar protein synthesis rates remained elevated over the 2–5 h postprandial period when compared with basal protein synthesis rates (from 0.020 ± 0.002%/h to 0.039 ± 0.004%/h; *P <* 0.001). In the BCKA and BCAA groups, myofibrillar protein synthesis rates decreased back to basal rates during the 2–5 h postprandial period (0.024 ± 0.005%/h and 0.024 ± 0.005%/h, respectively; *P >* 0.05). During the late (2–5 h) postprandial period, myofibrillar protein synthesis rates were higher in the MILK group when compared with the BCAA (*P* = 0.023) and BCKA groups (*P* = 0.023). A significant time × treatment interaction was also observed when comparing basal myofibrillar protein synthesis rates with the entire 5-h postprandial period (*P =* 0.003). Over the entire 5-h postprandial period (0–5 h), myofibrillar protein synthesis rates were significantly elevated above basal for all treatments (*P <* 0.005), but were significantly higher in the MILK group (0.040 ± 0.002%/h) compared with the BCKA (0.032 ± 0.003%/h; *P* = 0.023) and BCAA groups (0.032 ± 0.003%/h; *P* = 0.019), with no significant differences between the BCKA and BCAA groups (*P* > 0.05).

## Discussion

The present study demonstrated that ingestion of BCAAs, BCKAs, and MILK (providing an equivalent amount of BCAAs) significantly increased myofibrillar protein synthesis rates during the early (0–2 h) postprandial phase, with no differences observed between treatments. In the late (2–5 h) postprandial period myofibrillar protein synthesis rates remained elevated following MILK ingestion, with myofibrillar protein synthesis rates following BCAA and BCKA ingestion returning to baseline values.

It has been well established that ingestion of dietary protein strongly increases muscle protein synthesis rates (1, 2, 41). The postprandial increase in muscle protein synthesis rates has been attributed to the postprandial release of (essential) amino acids (3, 4, 42, 43), with the rise in circulating BCAAs and especially leucine being of particular relevance (16, 44–47). In line with previous work (10, 48, 49), we show a rapid postprandial increase in plasma (essential) amino acid concentrations following the ingestion of 30 g MILK, with plasma leucine concentrations reaching 294 ± 12 µmol/L within 30 min (Figure 5B). The postprandial rise in plasma amino acid availability resulted in a 111% increase in myofibrillar protein synthesis rates within the first 2 h after protein ingestion when compared with basal, postabsorptive myofibrillar protein synthesis rates (from 0.020 ± 0.002 to 0.042 ± 0.004%/h; Figure 8). In addition, postprandial myofibrillar protein synthesis rates assessed during the entire 5-h postprandial period were also higher following MILK ingestion when compared with baseline values (0.040 ± 0.002 compared with 0.020 ± 0.002%/h, respectively).

As the postprandial increase in muscle protein synthesis has been attributed largely to the postprandial increase in plasma BCAAs, we also assessed the impact of ingesting BCAAs only. Following ingestion of 6 g BCAAs, which equals the amount of BCAAs present in 30 g MILK, we observed a rapid rise in circulating BCAAs (Figure 6A), with plasma leucine concentrations reaching 2–3 fold higher concentrations when compared with MILK ingestion (Figure 5B). We also observed a strong, significant increase in myofibrillar protein synthesis rates within the first 2 h after BCAA ingestion (from 0.022 ± 0.002 to 0.044 ± 0.004%/h; Figure 8). This seems to be in line with previous work showing an increase in myofibrillar protein synthesis rate during a 2.5-h period after ingesting 3.4 g of free leucine (50). The rapid and marked increase in plasma BCAA availability after BCAA ingestion may have exceeded the maximal capacity to stimulate myofibrillar protein synthesis, so that during the 2-h postprandial period myofibrillar protein synthesis rates did not differ after ingestion of BCAA or MILK. Though this would be in line with the proposed muscle full effect (51, 52), these high myofibrillar protein synthesis rates could not be maintained following BCAA as opposed to MILK ingestion during the latter stages (2–5 h) of the postprandial period (Figure 8). These data suggest that besides the postprandial rise in plasma BCAA concentrations, other (essential) amino acids need to be provided to allow a more prolonged postprandial increase in muscle protein synthesis rate (53). Though the postprandial rise in muscle protein synthesis rate following protein ingestion can be initiated by the increased BCAA availability, it may be maintained only when sufficient other (essential) amino acids are provided to serve as precursors for de novo muscle protein synthesis. Alternatively, it could be speculated that ingestion of greater amounts (> 6 g) and/or a more sustained provision of BCAA are needed to prolong the elevation in muscle protein synthesis rates.

BCKAs are ketoanalogs of the 3 BCAAs and play an active role in BCAA metabolism, due to their rapid in vivo interconvertibility (by transamination) (26). As the transamination capacity is high in skeletal muscle (54), when BCKAs are transaminated into BCAAs, they may directly stimulate muscle protein synthesis. It has previously been shown that infusion with the BCKA α-ketoisocaproic acid stimulates muscle protein synthesis in neonatal pigs (33). However, the effect of BCKA ingestion on muscle protein synthesis rates in humans has never been assessed. Ingestion of 6 g BCKA, which was tolerated well in all subjects with no reported side effects, resulted in a strong postprandial increase in plasma BCKA concentrations (Figure 7). Concomitantly, we also observed a small but significant increase in plasma leucine concentrations (Figure 5B), suggesting that there is some, albeit limited, conversion of KIC to leucine in vivo in healthy humans. Despite only minimal changes in plasma BCAA or other amino acid concentrations following BCKA ingestion (Figure 6), we observed a rapid increase in myofibrillar protein synthesis rates during the early postprandial phase that did not differ from the early postprandial response following BCAA or MILK ingestion (Figure 8). However, similar to the postprandial response observed following BCAA ingestion, these elevated myofibrillar protein synthesis rates were not maintained during the latter postprandial phase (2–5 h). The observation that BCKA ingestion strongly increases (early) postprandial myofibrillar protein synthesis rates, despite a minimal rise in circulating plasma leucine concentrations, implies that either BCKA-derived BCAA (by transamination) are directly used within muscle to stimulate muscle protein synthesis or that alternative metabolites may be involved in the early postprandial stimulation of muscle protein synthesis. It has previously been shown that leucine metabolites, such as β-hydroxy-β-methylbutyrate, can stimulate myofibrillar protein synthesis (50, 55). In contrast to the ketoanalog of leucine, β-hydroxy-β-methylbutyrate cannot be converted back into leucine. In line with the minimal increase in plasma leucine concentrations following BCKA ingestion, our findings suggest that the stimulatory effect of BCKA ingestion on myofibrillar protein synthesis may work directly (KIC) and/or indirectly via conversion to β-hydroxy-β-methylbutyrate. Clearly, research is warranted to elucidate alternative metabolites and pathways that could be (at least partially) responsible for the stimulatory effect of BCKA ingestion on muscle protein synthesis. This will also yield important information on whether the anabolic properties of BCKA and BCAA involve separate pathways and, as such, may be combined to further increase postprandial muscle protein synthesis rates. Ultimately, net muscle protein accretion is determined by the balance between muscle protein synthesis and breakdown rates and it has been suggested that BCKA, with KIC in particular, has a profound impact on muscle protein breakdown. It appears that KIC, and not leucine per se, is primarily responsible for the inhibitory effect on muscle protein breakdown (56–59). This further supports the potential of BCKAs as an anabolic agent to stimulate muscle protein accretion.

BCKAs lack an amino group bound to the α-carbon in their molecular structure and, therefore, do not provide nitrogen. In support, transamination of the BCKAs into their respective BCAAs was likely responsible for the observed decline in plasma ammonia concentrations (Figure 4). The potential therapeutic value of BCKAs has been studied in several diseases and disorders, but appears particularly relevant for patients with CKD (25, 26). Patients with CKD are advised to adhere to low protein diets during certain stages of their disease (60), restricting them in their capacity to maintain muscle mass. The observed stimulatory effect of BCKA ingestion on early postprandial myofibrillar protein synthesis rates can be of particular relevance for this patient group as it may support them in preventing or attenuating the progressive loss of muscle mass without increasing nitrogen intake (34, 61). Furthermore, there are suggestions that a low protein diet improves the nutritional efficiency of BCKAs (26, 62). Therefore, future studies should look into the benefits of BCKA co-ingestion to stimulate postprandial muscle protein synthesis rates in patients with CKD ingesting a diet relatively low in dietary protein content.

In conclusion, ingestion of 6 g BCAAs, 6 g BCKAs, and 30 g MILK increases myofibrillar protein synthesis rates during the early postprandial phase (0–2 h) in vivo in healthy older males. The postprandial increase following the ingestion of 6 g BCAAs and BCKAs is short-lived, with higher myofibrillar protein synthesis rates only being maintained following the ingestion of an equivalent amount of intact milk protein.

## Supplementary Material

nqz120_Supplemental_Files

## Notes

The project was co-funded by Evonik Industries. The researchers are responsible for the study design, data collection and analysis, decision to publish, and preparation of the manuscript. The industrial partners have contributed to the project through regular discussion.

Data described in the manuscript (in de-identified form), code book, and analytic code will be made available upon request pending application and approval from the corresponding author.

Supplemental Figure 1 and Supplemental Methods are available from the “Supplementary data” link in the online posting of the article and from the same link in the online table of contents at https://academic.oup.com/ajcn/.

Abbreviations used: BCAA, branched-chain amino acid; BCKA, branched-chain ketoacid; CKD, chronic kidney disease; EAA, essential amino acid; FSR, fractional synthesis rate; HbA1c, glycated hemoglobin; iAUC, incremental AUC; KIC, α-ketoisocaproic acid; KIV, α-ketoisovalerate; KMV, α-keto-β-methylvalerate; MILK, milk protein; MPE, mole % excess; NEAA, nonessential amino acid; TAA, total amino acid.

## References

[1] Rennie MJ, Bohe J, Wolfe RR Latency, duration and dose response relationships of amino acid effects on human muscle protein synthesis. J Nutr. 2002;132:3225S–7S. 12368422 10.1093/jn/131.10.3225S

[2] Wolfe RR. Regulation of muscle protein by amino acids. J Nutr. 2002;132:3219S–24S. 12368421 10.1093/jn/131.10.3219S

[3] Bennet WM, Connacher AA, Scrimgeour CM, Smith K, Rennie MJ Increase in anterior tibialis muscle protein synthesis in healthy man during mixed amino acid infusion: Studies of incorporation of [1-13C]leucine. Clin Sci (Lond). 1989;76:447–54. 2714054 10.1042/cs0760447

[4] Bohe J, Low A, Wolfe RR, Rennie MJ Human muscle protein synthesis is modulated by extracellular, not intramuscular amino acid availability: A dose-response study. J Physiol. 2003;552:315–24. 12909668 10.1113/jphysiol.2003.050674 PMC2343318

[5] Atherton PJ, Smith K, Etheridge T, Rankin D, Rennie MJ Distinct anabolic signalling responses to amino acids in C2C12 skeletal muscle cells. Amino Acids. 2010;38:1533–9. 19882215 10.1007/s00726-009-0377-x

[6] Kimball SR, Jefferson LS. Signaling pathways and molecular mechanisms through which branched-chain amino acids mediate translational control of protein synthesis. J Nutr. 2006;136:227S–31S. 16365087 10.1093/jn/136.1.227S

[7] Churchward-Venne TA, Breen L, Phillips SM Alterations in human muscle protein metabolism with aging: Protein and exercise as countermeasures to offset sarcopenia. Biofactors. 2014;40:199–205. 24105883 10.1002/biof.1138

[8] Wall BT, Cermak NM, van Loon LJ Dietary protein considerations to support active aging. Sports Med. 2014;44:Suppl 2:S185–94. 25355192 10.1007/s40279-014-0258-7 PMC4213379

[9] Rennie MJ, Wilkes EA. Maintenance of the musculoskeletal mass by control of protein turnover: The concept of anabolic resistance and its relevance to the transplant recipient. Ann Transplant. 2005;10:31–4. 17037086

[10] Pennings B, Groen B, de Lange A, Gijsen AP, Zorenc AH, Senden JM, van Loon LJ Amino acid absorption and subsequent muscle protein accretion following graded intakes of whey protein in elderly men. Am J Physiol Endocrinol Metab. 2012;302:E992–9. 22338070 10.1152/ajpendo.00517.2011

[11] Moore DR, Churchward-Venne TA, Witard O, Breen L, Burd NA, Tipton KD, Phillips SM Protein ingestion to stimulate myofibrillar protein synthesis requires greater relative protein intakes in healthy older versus younger men. J Gerontol A Biol Sci Med Sci. 2015;70:57–62. 25056502 10.1093/gerona/glu103

[12] Baum JI, Kim IY, Wolfe RR Protein consumption and the elderly: What is the optimal level of intake? Nutrients. 2016;8:E359. 27338461 10.3390/nu8060359 PMC4924200

[13] Hoffer LJ, Bistrian BR. Appropriate protein provision in critical illness: A systematic and narrative review. Am J Clin Nutr. 2012;96:591–600. 22811443 10.3945/ajcn.111.032078

[14] Phillips SM. Current concepts and unresolved questions in dietary protein requirements and supplements in adults. Front Nutr. 2017;4:13. 28534027 10.3389/fnut.2017.00013 PMC5420553

[15] Katsanos CS, Kobayashi H, Sheffield-Moore M, Aarsland A, Wolfe RR A high proportion of leucine is required for optimal stimulation of the rate of muscle protein synthesis by essential amino acids in the elderly. Am J Physiol Endocrinol Metab. 2006;291:E381–7. 16507602 10.1152/ajpendo.00488.2005

[16] Rieu I, Balage M, Sornet C, Giraudet C, Pujos E, Grizard J, Mosoni L, Dardevet D Leucine supplementation improves muscle protein synthesis in elderly men independently of hyperaminoacidaemia. J Physiol. 2006;575:305–15. 16777941 10.1113/jphysiol.2006.110742 PMC1819434

[17] Wall BT, Hamer HM, de Lange A, Kiskini A, Groen BB, Senden JM, Gijsen AP, Verdijk LB, van Loon LJ Leucine co-ingestion improves post-prandial muscle protein accretion in elderly men. Clin Nutr. 2013;32:412–9. 23043721 10.1016/j.clnu.2012.09.002

[18] Devries MC, McGlory C, Bolster DR, Kamil A, Rahn M, Harkness L, Baker SK, Phillips SM Protein leucine content is a determinant of shorter- and longer-term muscle protein synthetic responses at rest and following resistance exercise in healthy older women: A randomized, controlled trial. Am J Clin Nutr. 2018;107:217–26. 29529146 10.1093/ajcn/nqx028

[19] Murphy CH, Saddler NI, Devries MC, McGlory C, Baker SK, Phillips SM Leucine supplementation enhances integrative myofibrillar protein synthesis in free-living older men consuming lower- and higher-protein diets: A parallel-group crossover study. Am J Clin Nutr. 2016;104:1594–606. 27935521 10.3945/ajcn.116.136424

[20] Jackman SR, Witard OC, Philp A, Wallis GA, Baar K, Tipton KD Branched-chain amino acid ingestion stimulates muscle myofibrillar protein synthesis following resistance exercise in humans. Front Physiol. 2017;8:390. 28638350 10.3389/fphys.2017.00390 PMC5461297

[21] Riccio E, Di Nuzzi A, Pisani A Nutritional treatment in chronic kidney disease: The concept of nephroprotection. Clin Exp Nephrol. 2015;19:161–7. 25319188 10.1007/s10157-014-1041-7

[22] Piccoli GB, Vigotti FN, Leone F, Capizzi I, Daidola G, Cabiddu G, Avagnina P Low-protein diets in CKD: How can we achieve them? A narrative, pragmatic review. Clin Kidney J. 2015;8:61–70. 25713712 10.1093/ckj/sfu125 PMC4310428

[23] Carrero JJ, Johansen KL, Lindholm B, Stenvinkel P, Cuppari L, Avesani CM Screening for muscle wasting and dysfunction in patients with chronic kidney disease. Kidney Int. 2016;90:53–66. 27157695 10.1016/j.kint.2016.02.025

[24] Houston DK, Nicklas BJ, Ding J, Harris TB, Tylavsky FA, Newman AB, Lee JS, Sahyoun NR, Visser M, Kritchevsky SB, Health ABCS. Dietary protein intake is associated with lean mass change in older, community-dwelling adults: The Health, Aging, and Body Composition (Health ABC) Study. Am J Clin Nutr. 2008;87:150–5. 18175749 10.1093/ajcn/87.1.150

[25] Shah AP, Kalantar-Zadeh K, Kopple JD Is there a role for ketoacid supplements in the management of CKD? Am J Kidney Dis. 2015;65:659–73. 25682182 10.1053/j.ajkd.2014.09.029

[26] Walser M. Role of branched-chain ketoacids in protein metabolism. Kidney Int. 1990;38:595–604. 2232501 10.1038/ki.1990.248

[27] Hall C, Grayson I. Genotoxicity and sub-chronic toxicity of MYOLUTION((R)) (branched chain keto acids). Regul Toxicol Pharmacol. 2017;90:133–43. 28888959 10.1016/j.yrtph.2017.09.004

[28] Walser M. Therapeutic aspects of branched-chain amino and keto acids. Clin Sci (Lond). 1984;66:1–15. 6360490 10.1042/cs0660001

[29] Weber FL Jr, Deak SB, Laine RA Absorption of keto-analogues of branched-chain amino acids from rat small intestine. Gastroenterology. 1979;76:62–70. 758150

[30] Swain LM, Shiota T, Walser M Utilization for protein synthesis of leucine and valine compared with their keto analogues. Am J Clin Nutr. 1990;51:411–5. 2309648 10.1093/ajcn/51.3.411

[31] May RC, Mitch WE. The metabolism and metabolic effects of ketoacids. Diabetes Metab Rev. 1989;5:71–82. 2649337 10.1002/dmr.5610050106

[32] Giordano M, Castellino P, Ohno A, Defronzo RA Differential effects of amino acid and ketoacid on protein metabolism in humans. Nutrition. 2000;16:15–21. 10674229 10.1016/s0899-9007(99)00211-7

[33] Escobar J, Frank JW, Suryawan A, Nguyen HV, Van Horn CG, Hutson SM, Davis TA Leucine and alpha-ketoisocaproic acid, but not norleucine, stimulate skeletal muscle protein synthesis in neonatal pigs. J Nutr. 2010;140:1418–24. 20534881 10.3945/jn.110.123042 PMC2903301

[34] Wang DT, Lu L, Shi Y, Geng ZB, Yin Y, Wang M, Wei LB Supplementation of ketoacids contributes to the up-regulation of the Wnt7a/Akt/p70S6K pathway and the down-regulation of apoptotic and ubiquitin-proteasome systems in the muscle of 5/6 nephrectomised rats. Br J Nutr. 2014;111:1536–48. 24502851 10.1017/S0007114513004091

[35] Alberti KG, Zimmet PZ Definition, diagnosis and classification of diabetes mellitus and its complications. Part 1: Diagnosis and classification of diabetes mellitus provisional report of a WHO consultation. Diabet Med. 1998;15:539–53. 9686693 10.1002/(SICI)1096-9136(199807)15:7<539::AID-DIA668>3.0.CO;2-S

[36] Bauer J, Biolo G, Cederholm T, Cesari M, Cruz-Jentoft AJ, Morley JE, Phillips S, Sieber C, Stehle P, Teta D et al. Evidence-based recommendations for optimal dietary protein intake in older people: A position paper from the PROT-AGE Study Group. J Am Med Dir Assoc. 2013;14:542–59. 23867520 10.1016/j.jamda.2013.05.021

[37] Abumrad NN, Rabin D, Diamond MP, Lacy WW Use of a heated superficial hand vein as an alternative site for the measurement of amino acid concentrations and for the study of glucose and alanine kinetics in man. Metabolism. 1981;30:936–40. 7022111 10.1016/0026-0495(81)90074-3

[38] Bergstrom J. Percutaneous needle biopsy of skeletal muscle in physiological and clinical research. Scand J Clin Lab Invest. 1975;35:609–16. 1108172

[39] Schierbeek H. Mass Spectrometry and Stable Isotopes in Nutritional and Pediatric Research. New Jersey: John Wiley & Sons, Inc; 2017, p. 56–61.

[40] Burd NA, Cermak NM, Kouw IW, Gorissen SH, Gijsen AP, van Loon LJ The use of doubly labeled milk protein to measure postprandial muscle protein synthesis rates in vivo in humans. J Appl Physiol (1985). 2014;117:1363–70. 25277738 10.1152/japplphysiol.00411.2014

[41] Gorissen SH, Remond D, van Loon LJ The muscle protein synthetic response to food ingestion. Meat Sci. 2015;109:96–100. 26021783 10.1016/j.meatsci.2015.05.009

[42] Tipton KD, Gurkin BE, Matin S, Wolfe RR Nonessential amino acids are not necessary to stimulate net muscle protein synthesis in healthy volunteers. J Nutr Biochem. 1999;10:89–95. 15539275 10.1016/s0955-2863(98)00087-4

[43] Volpi E, Kobayashi H, Sheffield-Moore M, Mittendorfer B, Wolfe RR Essential amino acids are primarily responsible for the amino acid stimulation of muscle protein anabolism in healthy elderly adults. Am J Clin Nutr. 2003;78:250–8. 12885705 10.1093/ajcn/78.2.250 PMC3192452

[44] Churchward-Venne TA, Breen L, Di Donato DM, Hector AJ, Mitchell CJ, Moore DR, Stellingwerff T, Breuille D, Offord EA, Baker SK et al. Leucine supplementation of a low-protein mixed macronutrient beverage enhances myofibrillar protein synthesis in young men: A double-blind, randomized trial. Am J Clin Nutr. 2014;99:276–86. 24284442 10.3945/ajcn.113.068775

[45] Norton LE, Layman DK. Leucine regulates translation initiation of protein synthesis in skeletal muscle after exercise. J Nutr. 2006;136:533S–7S. 16424142 10.1093/jn/136.2.533S

[46] Escobar J, Frank JW, Suryawan A, Nguyen HV, Kimball SR, Jefferson LS, Davis TA Regulation of cardiac and skeletal muscle protein synthesis by individual branched-chain amino acids in neonatal pigs. Am J Physiol Endocrinol Metab. 2006;290:E612–21. 16278252 10.1152/ajpendo.00402.2005

[47] Devries MC, McGlory C, Bolster DR, Kamil A, Rahn M, Harkness L, Baker SK, Phillips SM Leucine, not total protein, content of a supplement is the primary determinant of muscle protein anabolic responses in healthy older women. J Nutr. 2018;148(7):1088–95. 29901760 10.1093/jn/nxy091

[48] Trommelen J, Kouw IWK, Holwerda AM, Snijders T, Halson SL, Rollo I, Verdijk LB, van Loon LJC Presleep dietary protein-derived amino acids are incorporated in myofibrillar protein during postexercise overnight recovery. Am J Physiol Endocrinol Metab. 2018;314:E457–67. 28536184 10.1152/ajpendo.00273.2016

[49] Koopman R, Crombach N, Gijsen AP, Walrand S, Fauquant J, Kies AK, Lemosquet S, Saris WH, Boirie Y, van Loon LJ Ingestion of a protein hydrolysate is accompanied by an accelerated in vivo digestion and absorption rate when compared with its intact protein. Am J Clin Nutr. 2009;90:106–15. 19474134 10.3945/ajcn.2009.27474

[50] Wilkinson DJ, Hossain T, Hill DS, Phillips BE, Crossland H, Williams J, Loughna P, Churchward-Venne TA, Breen L, Phillips SM et al. Effects of leucine and its metabolite beta-hydroxy-beta-methylbutyrate on human skeletal muscle protein metabolism. J Physiol. 2013;591:2911–23. 23551944 10.1113/jphysiol.2013.253203 PMC3690694

[51] Atherton PJ, Etheridge T, Watt PW, Wilkinson D, Selby A, Rankin D, Smith K, Rennie MJ Muscle full effect after oral protein: Time-dependent concordance and discordance between human muscle protein synthesis and mTORC1 signaling. Am J Clin Nutr. 2010;92:1080–8. 20844073 10.3945/ajcn.2010.29819

[52] Bohe J, Low JF, Wolfe RR, Rennie MJ Latency and duration of stimulation of human muscle protein synthesis during continuous infusion of amino acids. J Physiol. 2001;532:575–9. 11306673 10.1111/j.1469-7793.2001.0575f.x PMC2278544

[53] Wolfe RR. Branched-chain amino acids and muscle protein synthesis in humans: Myth or reality? J Int Soc Sports Nutr. 2017;14:30. 28852372 10.1186/s12970-017-0184-9 PMC5568273

[54] Suryawan A, Hawes JW, Harris RA, Shimomura Y, Jenkins AE, Hutson SM A molecular model of human branched-chain amino acid metabolism. Am J Clin Nutr. 1998;68:72–81. 9665099 10.1093/ajcn/68.1.72

[55] Wilkinson DJ, Hossain T, Limb MC, Phillips BE, Lund J, Williams JP, Brook MS, Cegielski J, Philp A, Ashcroft S et al. Impact of the calcium form of beta-hydroxy-beta-methylbutyrate upon human skeletal muscle protein metabolism. Clin Nutr. 2018;37(6 Pt A):2068–75. 29097038 10.1016/j.clnu.2017.09.024 PMC6295980

[56] Francois G, Blanc M, Calderon A, Rose F Effect of leucine or ketoleucine on nitrogen metabolism in postoperative patients receiving energy substrate. Clin Nutr. 1984;3:99–101. 16829442 10.1016/s0261-5614(84)80007-2

[57] Francois G, Calderon A, Rose F, Blanc M, Lena P Inhibition of postoperative muscular proteolysis by sodium alpha-ketoisocaproate: Does a dose-effect relation exist?. Ann Fr Anesth Reanim. 1985;4:351–4. 4037442 10.1016/s0750-7658(85)80104-0

[58] Sapir DG, Stewart PM, Walser M, Moreadith C, Moyer ED, Imbembo AL, Rosenshein NB, Munoz S Effects of alpha-ketoisocaproate and of leucine on nitrogen metabolism in postoperative patients. Lancet. 1983;1:1010–4. 6133059 10.1016/s0140-6736(83)92643-0

[59] Tischler ME, Desautels M, Goldberg AL Does leucine, leucyl-tRNA, or some metabolite of leucine regulate protein synthesis and degradation in skeletal and cardiac muscle? J Biol Chem. 1982;257:1613–21. 6915936

[60] Kalantar-Zadeh K, Fouque D. Nutritional management of chronic kidney disease. N Engl J Med. 2017;377:1765–76. 29091561 10.1056/NEJMra1700312

[61] Jahn H, Rose F, Schmitt R, Melin G, Schohn D, Comte G, Schaetzel S Protein synthesis in skeletal muscle of uremic patients: Effect of low-protein diet and supplementation with ketoacids. Miner Electrolyte Metab. 1992;18:222–7. 1465063

[62] Kang CW, Tungsanga K, Walser M Effect of the level of dietary protein on the utilization of alpha-ketoisocaproate for protein synthesis. Am J Clin Nutr. 1986;43:504–9. 3962903 10.1093/ajcn/43.4.504

