# Supplementary material for: Branched-chain amino acid and branched-chain ketoacid ingestion increases muscle protein synthesis rates in vivo in older adults: a double-blind, randomized trial
Source: Am J Clin Nutr. 2019 Jun 28;110(4):862–72. doi: 10.1093/ajcn/nqz120 (PMC6766442; doi:10.1093/ajcn/nqz120)
Supplement: nqz120_Supplemental_Files [file nqz120_supplemental_files.zip › SUPPLEMENTAL METHODS.docx]

**SUPPLEMENTARY METHODS**

*Plasma and muscle tissue analysis*

Plasma glucose and insulin concentrations were analyzed using commercially available kits (ref. no. A11A01667, Glucose HK CP, ABX Diagnostics, Montpellier, France; and ref. no. HI-14K, Millipore, St. Louis, MO, respectively). Plasma ammonia was measured enzymatically with glutamate dehydrogenase performed on a Cobas Fara automatic analyser (Roche, Basel, Switzerland) [1]. Quantification of plasma keto- and amino acid concentrations was performed using ultra-performance liquid chromatograph mass spectrometry (UPLC-MS; ACQUITY UPLC H-Class with QDa; Waters, Saint-Quentin, France). 50 µl of blood plasma was deproteinized using 100 µL of 10 % SSA with 50 µM of MSK-A2 internal standard (Cambridge Isotope Laboratories, Massachusetts, USA). Subsequently, 50 µL of ultra-pure demineralized water was added and samples were centrifuged (15 min at 14000 rpm). After centrifugation, 10 µL of supernatant was added to 70 µL of Borate reaction buffer (Waters, Saint-Quentin, France). In addition, 20 µL of AccQ-Tag derivatizing reagent solution (Waters, Saint-Quentin, France) was added after which the solution was heated to 55 °C for 10 min. An aliquot of 1 µL was injected and measured using UPLC-MS. HbA_1c_ content was determined in venous blood samples by high-performance liquid chromatography (Bio-Rad Variant II, Munich, Germany). For plasma L-[ring-^13^C_6_]-phenylalanine enrichment measurements, plasma phenylalanine was derivatized to the tert-butyldimethylsilyl (TBDMS) derivative with N-tert-butyldimethylsilyl-N-methyltrifluoroacetamide (MTBSTFA), and the ^13^C enrichments were determined by electron ionization gas chromatography-mass spectrometry (GC-MS; Agilent 7890A GC/5975C MSD; Agilent Technologies) using selected ion monitoring of masses 336 and 342 for unlabeled and labeled (ring-^13^C_6_) phenylalanine, respectively. We applied standard regression curves in all isotopic enrichment analyses to assess linearity of the mass spectrometer and to control for loss of tracer. Through the addition of an internal standard phenylalanine (m+10), concentrations of phenylalanine were determined in the same run. Phenylalanine enrichments were corrected for the natural level of ^13^C isotopes. Mixed plasma proteins were isolated from blood samples by using perchloric acid (PCA) to a final concentration of 2 %. Samples were centrifuged at 1000 *g* at 4 °C for 10 min, and the supernatants were removed. The mixed plasma protein pellet was washed 3 times with 2 % PCA and dried. Amino acids were liberated by adding 6 M HCl and were heated at 120 °C for 15–18 h. Thereafter, the enrichments in hydrolyzed mixed plasma protein samples were assessed using the same procedures as the muscle protein-bound samples (described below).

Myofibrillar protein enriched fractions were isolated from ~60 mg of wet muscle tissue by hand-homogenizing on ice using a pestle in a standard extraction buffer (7 μL·mg^-1^). The samples were spun at 800 *g* and 4 ºC for 15 min. The pellet was washed with 500 µL ddH2O and centrifuged at 800 *g* and 4 ºC for 10 min. The myofibrillar protein was solubilized by adding 1 mL of 0.3 M NaOH and heating at 50 ºC for 30 min with vortex mixing every 10 min. Samples were centrifuged at 9500 *g* and 4 ºC for 5 min, the supernatant containing the myofibrillar proteins was collected and the collagen pellet was discarded. Myofibrillar proteins were precipitated by the addition of 1 mL of 1 M PCA and spinning at 1000 *g* and 4 ºC for 10 min. The myofibrillar protein was washed twice with 70 % ethanol and hydrolyzed overnight in 2 mL of 6 M HCL at 110 ºC. The free amino acids from the hydrolyzed myofibrillar protein pellet were dried under a nitrogen stream while being heated to 120 ºC. The free amino acids were then dissolved in 25 % acetic acid solution, passed over cation exchange AG 50W-X8 resin columns (mesh size: 100-200, ionic form: hydrogen; Bio-Rad Laboratories, Hercules, CA), washed 5 times with water and finally eluted with 2 M NH_4_OH. To determine myofibrillar protein L-[ring*-*^13^C_6_]-phenylalanine enrichments, the purified amino acids were first converted into N-ethoxycarbonyl ethyl ester derivatives with ethyl chloroformate (ECF). The derivatives were then measured by GC-C-IRMS (Thermo Fisher Scientific Delta V, Bremen, Germany) using a DB-17MS-column (30 m x 0.25 mm x 0.5 µm; Agilent J+W scientific GC column, Santa Clara, CA, USA) and monitoring of ion masses 44, 45, and 46. Standard regression curves were applied to assess the linearity of the mass spectrum and to account for isotopic fractionation.

**REFERENCES**

1. Janssen MAvB, C. L. H., van Leeuwen, P. A. M.; Soeters, P. B. : **The determination of ammonia in plasma and whole blood.** *Advances in Ammonia Metabolism and Hepatic Encephalopathy, ed Soeters, P B, Wilson, J H P, Meijer, A J & Holm, R* 1988**:**587-592.
