# Supplementary material for: Branched-chain amino acid and branched-chain ketoacid ingestion increases muscle protein synthesis rates in vivo in older adults: a double-blind, randomized trial
Source: Am J Clin Nutr. 2019 Jun 28;110(4):862–72. doi: 10.1093/ajcn/nqz120 (PMC6766442; doi:10.1093/ajcn/nqz120)
Supplement: nqz120_Supplemental_Files [file nqz120_supplemental_files.zip › Supplementary Figure 1.pptx]

## Slide 1
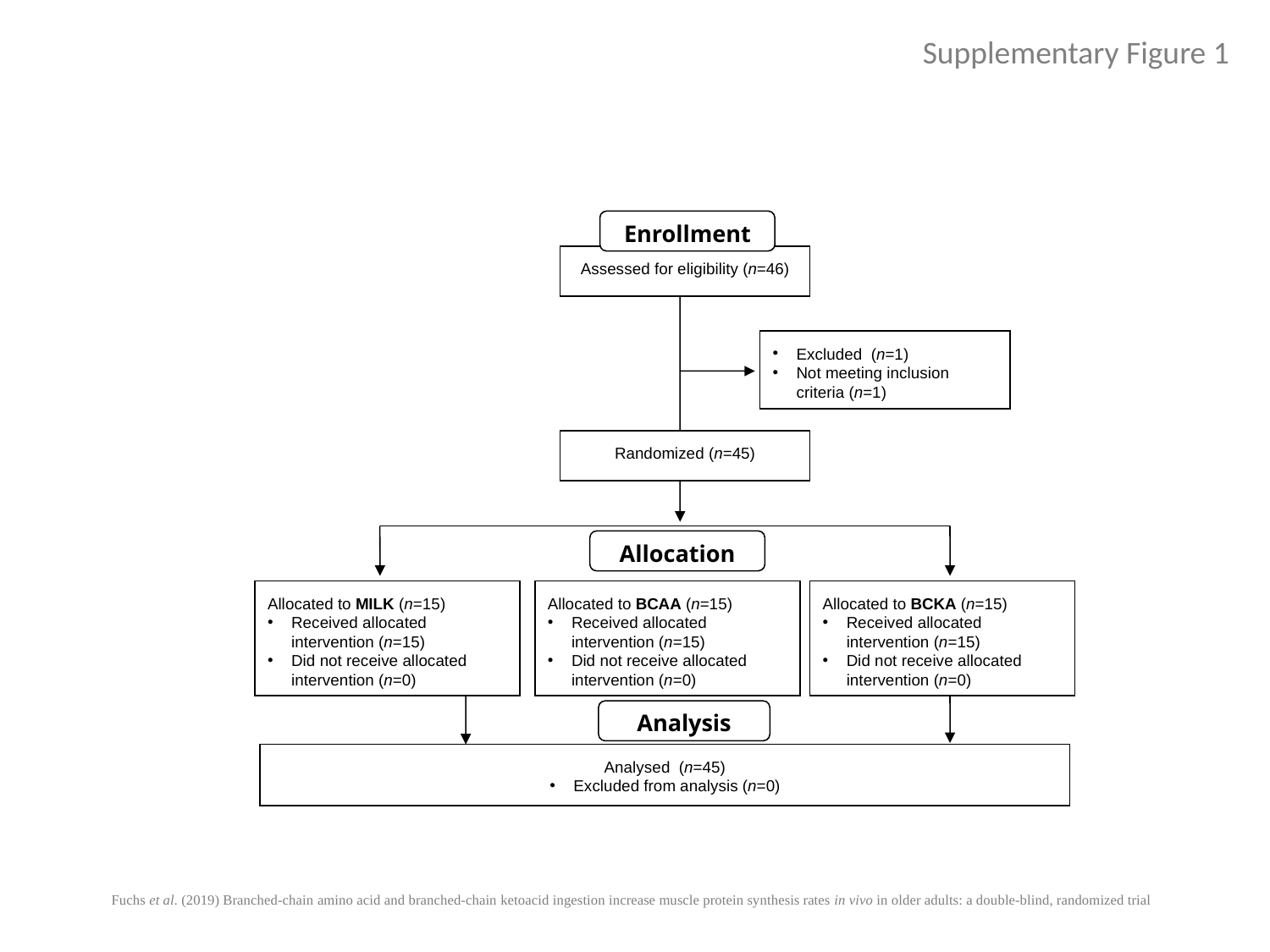

Supplementary Figure 1
Enrollment
Assessed for eligibility (n=46)
Excluded (n=1)
Not meeting inclusion criteria (n=1)
Randomized (n=45)
Allocation
Allocated to BCAA (n=15)
Received allocated intervention (n=15)
Did not receive allocated intervention (n=0)
Allocated to BCKA (n=15)
Received allocated intervention (n=15)
Did not receive allocated intervention (n=0)
Analysis
Analysed (n=45)
Excluded from analysis (n=0)
Allocated to MILK (n=15)
Received allocated intervention (n=15)
Did not receive allocated intervention (n=0)
Fuchs et al. (2019) Branched-chain amino acid and branched-chain ketoacid ingestion increase muscle protein synthesis rates in vivo in older adults: a double-blind, randomized trial
